# Uncommon presentations of an uncommon entity: OHVIRA syndrome with hematosalpinx and pyocolpos

**Published:** 2017-09

**Authors:** Z Sleiman, T Zreik, R Bitar, R Sheaib, A Al Bederi, V Tanos

**Affiliations:** Lebanese American University, Department of Obstetrics and Gynecology, Beirut, Lebanon; Tiba IVF center, Hilla, Irak; St. George’s Medical School, Nicosia University, Nicosia, Cyprus

**Keywords:** OHVIRA, pyocolpos, hematosalpinx, hysteroscopy, laparoscopy, MRI, ultrasound

## Abstract

Müllerian malformations result from defective fusion of the Müllerian ducts during development of the female reproductive system. The least common form of these malformations is Herlyn-Werner-Wunderlich syndrome characterized by obstructed hemivagina and ipsilateral renal anomaly (OHVIRA). The most common presentation of this syndrome is a mass secondary to hematocolpos, pain, and dysmenorrhea. Clinical diagnosis is very challenging and requires imaging studies in which ultrasound and MRI play an essential role in the diagnosis, classification and treatment plan.

We report two cases of this syndrome, featuring two very rare clinical presentations: hematosalpinx and pyocolpos. The clinical course of the pathology is not standard and each patient is treated accordingly.

## Introduction

A didelphic uterus with an obstructed hemivagina and ipsilateral renal agenesis (OHVIRA) is a rare congenital anomaly constituting 0.16–10% of all Müllerian duct abnormalities described only in case reports in medical literature (Noviella et al., 2017). It is most commonly diagnosed in puberty due to pelvic and abdominal pain, worsening dysmenorrhea, a pelvic mass, and an ipsilateral renal agenesis. More rarely it can present in neonates or adults, with primary infertility, pyometra, urinary obstruction and ischiorectal swelling (Raju et al., 2015). Due to its rarity, it can cause a delay in diagnosis which increases the risk of complications such as endometriosis, pyocolpos and infertility (Cox and Ching, 2012; Jung et al., 2017). The condition is most commonly diagnosed by imaging with ultrasound or computed tomography (CT) scan, although more recently magnetic resonance imaging (MRI) has emerged as the most sensitive diagnostic method (Yilmaz et al., 2017) Treatment is carried out through excision of the vaginal septum, with good results.

## Case presentation

We present two cases of OHVIRA syndrome with two different rare clinical presentations.

**Figure 1:**
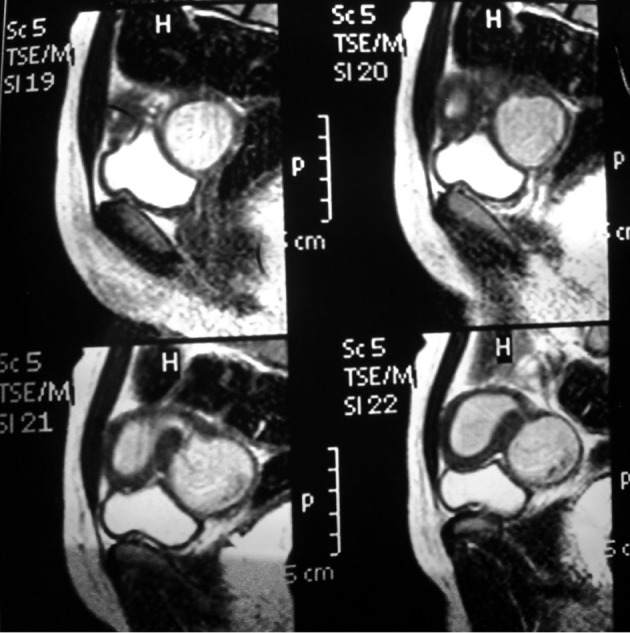
— MRI images showing the hematometra along with the hematocolpos

The first case is a 16 year-old Iraqi patient referred to our clinic for chronic pelvic pain and progressive painful distention of the lower abdomen. Her medical history was uneventful and menarche was at the age of 12. The clinical exam showed normal external genitalia. The vagina was occupied by a large soft painful mass and a normal cervix was identified on the speculum exam. The pelvic ultrasound showed two endometrial cavities, the left side being dilated with a fluid collection. It also showed a para-ovarian left pelvic mass suggesting a hematosalpinx, and a vaginal collection of 6x4 cm2. Abdominal ultrasound showed the absence of a left kidney. Subsequently, MRI was performed to better characterize the pelvic anatomy and better identify the anatomic location of the pelvic fluid collections. A didelphic uterus was demonstrated. The right endometrial cavity appeared normal along with the right cervix. On the left side, it showed a hematosalpinx. The endometrial cavity was distended, and contiguous with the left obstructed hemivagina (Fig. 1).

**Figure 2:**
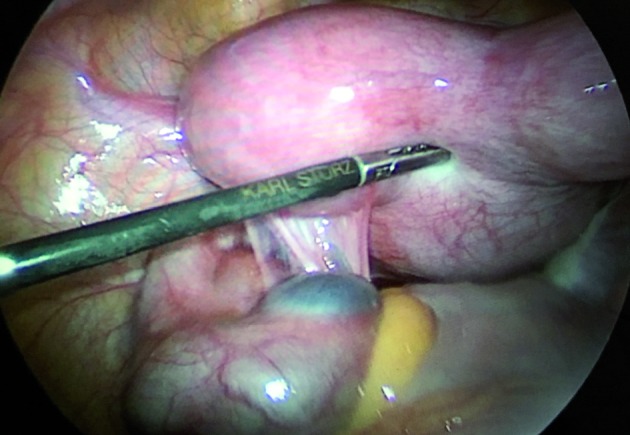
— Laparoscopic image showing the hematocolpos, dilated left horn, and left hematosalpinx.

The patient was scheduled for surgical treatment. The laparoscopy showed a bicornuate uterus with a dilated left horn, and an ipsilateral hematosalpinx. The posterior wall of the vagina was bulging into the peritoneal cavity. No endometriosis implants were identified (Fig. 2).

The second case is a 20 year-old Iraqi patient, who presented to our clinic for a 2 years history of primary infertility and recent dyspareunia. History was uneventful and menarche was at the age of 13.

**Figure 3:**
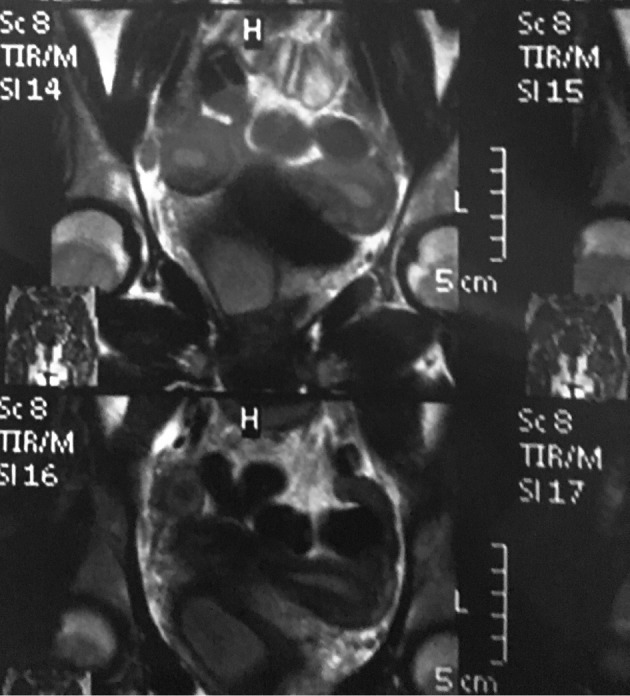
— MRI image showing the bicornuate uterus and the hematocolpos

The clinical exam showed normal external genitalia, and a vaginal fluctuant painful mass obliterating the right part of the vagina. The speculum exam showed a normal cervix. Ultrasound showed a didelphic uterus with a 3x4 cm2 homogenous collection in the vagina. Abdominal ultrasound showed the absence of a right kidney. An OHVIRA syndrome was suspected and the patient was sent for an MRI for further evaluation. The MRI showed a bicornuate uterus, with two crevices, the right one communicating with a right vaginal collection due to a longitudinal hemi vaginal septum (Fig. 3).

**Figure 4:**
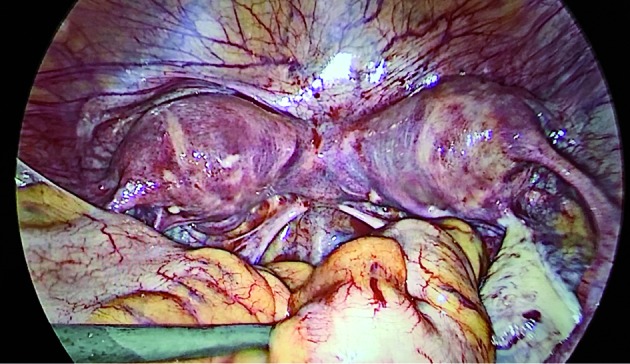
— Laparoscopic image showing a symmetrical bicornuate uterus.

The laparoscopy showed a bicornuate uterus and normal adnexa. Unlike the first case, the vaginal collection was not clearly identified (Fig. 4).

**Figure 5:**
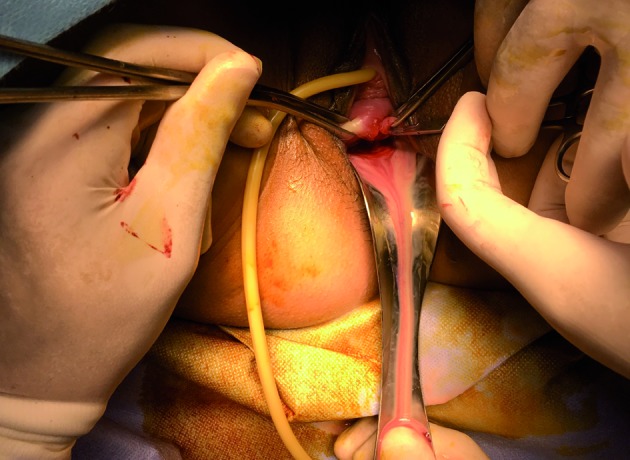
— Drainage of the pyocolpos.

Whereas in the first case we proceeded with a left salpingectomy, we treated both vaginal collections with the same approach: vaginoplasty with excision of the vaginal septum. In the first case, the drainage of the collection showed a dark hematic thick fluid. Surprisingly, excision of the right vaginal septum in second case drained a purulent discharge. Cultures grew out sensitive Escherichia coli (Fig. 5).

**Figure 6:**
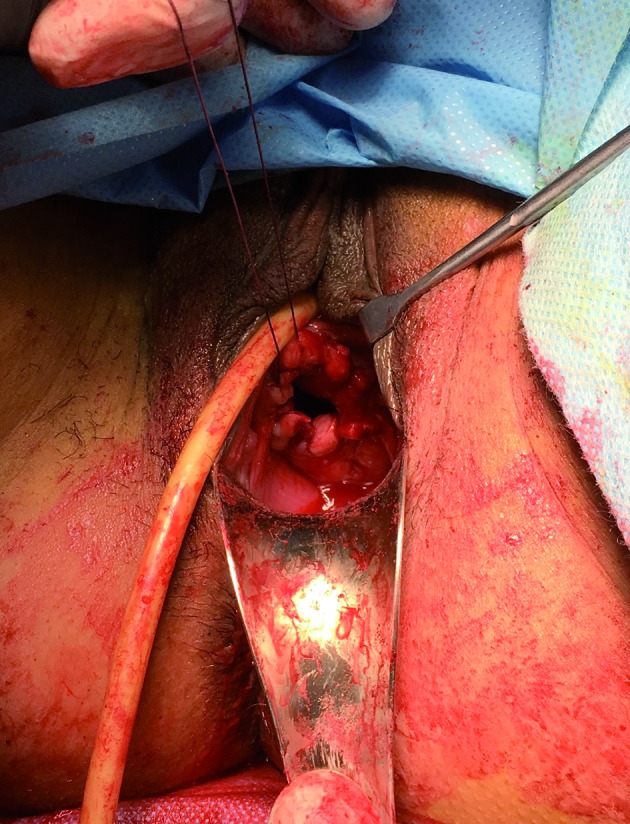
— Vaginoplasty and marsupialization of the vaginal edges.

In both cases, we performed a marsupialization of the vaginal edge after a large excision of the septum to prevent re-obstruction and the recurrence of collection (Fig. 6).

## Discussion

Müllerian duct abnormalities cover a wide range of developmental anomalies, resulting from non- development, defective fusion, or defects in regression of the septum during fetal development. Herlyn-Werner-Wunderlich syndrome (HWW) is a rare disease comprising a triad of didelphic uterus, obstructed hemivagina and renal agenesis (most often ipsilateral) (AFS classification, 1988). It is classified as U3bc2V2 following the comprehensive new classification of the ESHRE/ ESGE (Grimbizis et al., 2013). Vertical fusion defects such as transverse vaginal septum can develop anywhere in the vagina. The septum is commonly localized at the junction of the upper and middle third of vagina. Complete obstruction is a rare variant regarding to commonly septal fenestration (Jindal et al., 2009). Both cases presented a vaginal septum emerging from the cervical ostium, extending throughout whole vaginal length and ending at the level of the hymen.

A mass secondary to hematocolpos due to retained menstrual flow is a characteristic clinical finding while long standing dysmenorrhea and vaginal pain are the most frequent symptoms (Aydin et al., 2014). In incomplete obstruction, as in our patients, the diagnosis can be delayed, as there is one hemivagina which is patent, allowing for menstrual blood to exit, while the other side is obstructed; this eventually leads to a large hematocolpos (Wozniakowska et al., 2014).

It has been postulated that reversed menstruation increases the risk of endometriosis, pelvic adhesions, infertility and pelvic inflammatory disease (Jindal et al., 2009). Nevertheless, no clear evidence was found regarding tubo-ovarian abscess or endometriosis associated with the OHVIRA syndrome, except few case reports describing the rare complication of pyocolpos (Dhar et al., 2011).

The first patient, although 4 years younger than the second, was expected to experience less number of menstruations, yet she had larger hematocolpos and hematosalpinx, demonstrating the individual response to vaginal expansibility to accommodating large volume of menses and to change the course of the pathology.

Both patients had intercourse however only the older patient with a smaller collection without any history of fever or shivering, presented pyosalpinx, probably due to an occasional low immunity and consequent ascending E. Coli infection. A larger number of patients are necessary to determine factors that affect the course of the pathology in term of blood amount and infection risk. However, infection and pyocolpos risk is higher once intercourse is reported.

Ultrasound and MRI are the modalities of choice for the diagnosis and surgical planning of OHVIRA. Although ultrasound can be used to diagnose this condition, MRI is superior to ultrasound by allowing better uterine shape characterization and anatomic relationships of adjacent organs, given its multiplanar capabilities and wider field of view.

Despite its high sensitivity, MRI couldn’t identify preoperatively the purulent character of the collection in the second case. In fact we kept a low index of suspicion since the patient never reported episodes of high fever or shivering. We conclude that diagnosis of infected hematocolpos should be kept in mind even in the absence of the infection clinical manifestations such as fever and chills.

Most surgeons completely excise the vaginal septum and then both normal vaginal mucosal edges are sutured together over the defect. Others prefer to marsupialize the edges of the septum (Tug et al., 2015). Large excision of the septum and marsupialization of the edges were performed in both our cases.

We performed laparoscopic salpingectomy in the case of associated hematosalpinx. As recommended by Grimbizis et al. (2016) in the case of complex obstructive anomalies, we also performed diagnostic laparoscopy in the second case for better description of the pathology. Nevertheless, one can ask about the benefit of laparoscopy and hysteroscopy in the case of OHVIRA syndrome, well characterized on the MRI and not associated with tubal pathology. Even though some authors reported the use of hysteroscopy for incision of the vaginal septum, as a hymen conservative technique (Tug et al., 2015) it remains of no added value for diagnostic purposes. Others also used laparoscopy for drainage of hematocolpos in order to postpone the septum incision, but as for hysteroscopy, laparoscopy doesn’t give any further information over the MRI. We suggest that good clinical assessment followed by ultrasound and MRI is enough as diagnostic tools. In the absence of associated tubal pathology, surgeons can proceed straight forward to the excision of the septum.

Women with uterus didelphys have a high pregnancy rate of 80% (Heinonen, 2000) but with elevated rates of premature delivery (22%) and abortion (74%); cesarean section is necessary in over 80% of patients because breech position has a higher incidence (Sugiura-Ogasawara et al., 2013).

Our patients were followed up for a year after surgery, there was no recurrence of the collections, but none of them got pregnant.

## Conclusion

OHVIRA should be suspected in menstruating women with cyclic pelvic pain, vaginal mass, and unilateral renal agenesis. The syndrome can present with associated hematosalpinx or pyocolpos that can happen independently of the pathology course. In the presence of MRI characterization, laparoscopy has a limited role as a diagnostic tool, but remains necessary for treatment of associated tubal pathology.

## Conflict of interest

 Dr. Zaki Sleiman is a consultant for Karl Storz endoscope. He declares that his relation with the company mentioned above has no impact upon the scientific value and the content of the submitted.
